# A New Way of Airline Traffic Prediction Based on GCN-LSTM

**DOI:** 10.3389/fnbot.2021.661037

**Published:** 2021-12-10

**Authors:** Jiangni Yu

**Affiliations:** School of Economics and Management, Beijing University of Posts and Telecommunications, Beijing, China

**Keywords:** graph convolutional network, long short term memory network, flow, airlines, predict

## Abstract

With the development of society and the improvement of people's material level, more and more people like to travel by airplane. If we can predict the passenger flow of an airline in advance, it can be used as an important decision-making basis for its flight route planning, crew scheduling planning and ticket price formulation in the process of management and operation. However, due to the high complexity of aviation network, the existing traffic prediction methods generally have the problem of low prediction accuracy. In order to overcome this problem, this paper makes full use of graph convolutional neural network and long—short memory network to construct a prediction system with short—term prediction ability. Specifically, this paper uses the graph convolutional neural network as a feature extraction tool to extract the key features of air traffic data, and solves the problem of long term and short term dependence between data through the long term memory network, then we build a high-precision air traffic prediction system based on it. Finally, we design a comparison experiment to compare the algorithm with the traditional algorithms. The results show that the algorithm we proposed in this paper has a higher accuracy in air flow prediction according to the lower loss function value.

## 1. Introduction

In recent years, as an important industry in national economic and social development and an advanced mode of transportation, the demand for civil aviation passenger transport has been growing rapidly along with the rapid development of national economy and the substantial increase of people's income. Air fare has always been the focus of attention because it is related to the development of civil aviation industry, the profitability of airlines and the vital interests of passengers. Pricing strategies of airlines are mostly based on revenue management theory (Donovan, 2005; Klein et al., 2020), in which air traffic forecasting plays an important role. The forecast results can be used to dynamically adjust the ticket prices, so that the airlines can get the maximum benefits (Diego and Sang-Yeob, 2012). However, the pricing mechanism of each airline is complex, and the real-time ticket price is constantly changing under the influence of many factors, which has the characteristics of trend, randomness and volatility. Therefore, how to forecast air passenger flow accurately and reasonably has become an important content of air transport management in China.

Air passenger flow prediction has been studied at home and abroad. In Etzioni et al. (2003), an ensemble learning binary classification algorithm by Hamlet, is proposed based on Q-learning. Hamlet applies rule learning, reinforcement learning and time series techniques, and combines their results through superposition generalization to produce the final decision. The accuracy can reach 74.5%. There is still much room for improvement in traffic prediction. Riedel and Gabrys (2003) used autoregressive model (AR) to realize the prediction of air traffic. An AR is the regression of a variable relative to itself, using past observations to make predictions about current values. Moving average model (MA) is a regression model based on past forecasting errors. Sickles et al. (1998) proposed a model ARMA that combines autoregressive model and moving average model. ARIMA (autoregressive integrated moving average) (Li et al., 2018) and Bayesian structured time series Bayesian structured time series (BSTS) model (Kulkarni et al., 2018; Madhavan et al., 2020) are used to forecast the air passenger and cargo demand of the Indian aviation industry. Wang et al. (2013) used gray scale model to forecast passenger flow, applied a function to the observation data set, converted it into a monotonically increasing data set, and obtained the forecast results. Carmona-Benítez and Nieto (2020) proposed a new damping trend gray model (DTSM) based on dynamic seasonal damping factor, which is used to predict airline passenger demand (PAX) in the air transport industry. This model is called Sarima Damped Trend Gray Prediction Model (SDTGM), which can effectively improve the accuracy of prediction.

The classical time series model is based on the assumption that there is a linear relationship between the past and the future value, but the time series model to predict the traffic demand data must have correlation, because the passenger flow is affected by various factors change, trend and randomness and volatility characteristics, which will also affect the forecast of air flow. Causal models attempt to establish an explanatory equation that adequately describes the observation as the output of one or more underlying causative factors. Liu and Li (2012) proposed to use the probability logarithmic model to predict passenger flow, establish factors that can explain past demands, and then use the model to provide predicted values. Logit models, such as multinomial models, nested models and cross-nested models, are widely used in demand prediction of passenger selection models Leng et al. (2015).

It is a good trend to apply machine learning technology to solve nonlinear time series problems. Various machine learning algorithms have been studied and deployed. Neural Network model Tsai et al. (2009); Babai et al. (2020); Yustiawan et al. (2021) is widely used in long-term demand prediction. Grimme et al. (2020) use them for short-term demand forecasting. They compared two advanced formulas, MTUNN and PENN, with the general multilayer perceptron model. Wei and Chen (Chen et al., 2020) used hybrid neural network model to predict passenger flow in rapid transit systems. A Bayesian state-space model can be created by generating the current state matrix from the observed data in the time series (Zhang et al., 2020). Support vector machine (SVM) is regarded as a classification tool, and support vector regression aims to identify and optimize the error range of regression. Jiang et al. (2015) applied gray SVM combined with empirical mode decomposition (EMD) to high-speed railway passenger flow prediction. Xie et al. (2013) used EMD to model passenger flow prediction of airport terminals by least squares support vector machine. Weng et al. (2019) applied the hybrid model combining LSTM and Light TGBM to air ticket sales prediction. Artificial intelligence or machine learning models are computation-intensive self-learning algorithms that iteratively modify and fine-tune the interpretation model through the evaluation results, and reduce the margin of error. However, the above method based on machine learning is based on the historical data of each air station forecast, this method does not take into account the impact of ticket prices and the ridership flow of other stations and the current ridership flow of the station, so the prediction performance is poor.

Graphic Convolutional Neural Network (GCN), as a Neural Network that can extract unstructured data, which has attracted a lot of attention in solving the relationship between adjacent points (Wei et al., 2019; He et al., 2020). In view of the low accuracy of air passenger flow prediction and the trend, randomness and volatility of air traffic affected by many factors, we built a graph convolution-long short-term memory model based on graph convolutional neural network and the long short-term memory (LSTM) neural network. In this model, the GCN is used to map the features of the data set. Then the LSTM model are used to process the matrix data set, and the fare prediction is realized. The method considers the different effects of various factors on the ticket price, and combines the trend and fluctuation characteristics of the ticket price. The experimental results show that the graph convolution model of long memory and short memory can predict the air ticket price well. Our contributions are summaried as:

1. In order to improve the feature extraction ability of aviation data, we have a deep understanding of the data characteristics of aviation data. By constructing a GCN feature extractor, we can transform non-European spatial data into concise and efficient features. This method improves the feature expression ability of the data.
2. In order to solve the problem of long term and short term dependence of time series data, in this paper, we introduce the LSTM network to solve the data dependence of samples through logical gating unit, and further improve the performance of prediction.
3. The air flow prediction scheme based on GCN-LSTM shows excellent performance on the existing aviation data. The experimental results show that the prediction performance of the proposed algorithm is obviously better than that of other existing algorithms.

The remaining part of this paper is organized as follows. The main contribution of this paper is descripted in section 2. In section 3, two comparative experiments are used to prove the effectiveness of the algorithm. And conclusions are drawn in section 4.

## 2. Main Result

**Figure 1:**
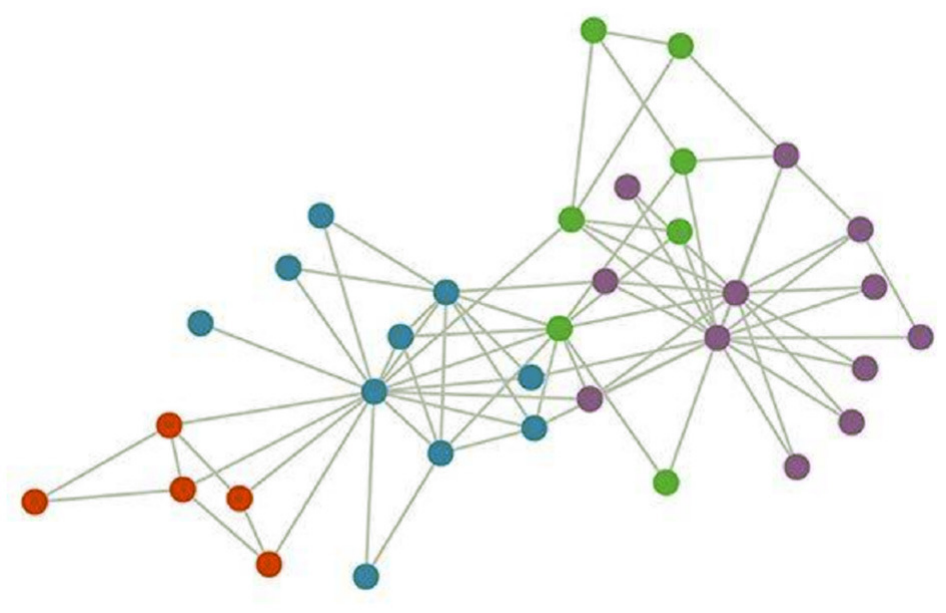
The distribution of the stations.

In airlines, the spatial distribution of aircraft stations is a non-euclidean structure, that is, the number of stations around each station is uncertain, and even if two stations are adjacent, they may not actually communicate with each other, resulting in no spatial relationship between their traffic. Therefore, traditional convolutional neural network (CNN) cannot accurately obtain their spatial information. At this point, multiple sites can be abstracted into A graph (see Figure 1). Features are extracted from the original input data to obtain the result of feature mapping of multiple channels. The intercommunication relationship between each site is represented by adjacency matrix A.

### 2.1. The Definition of the Problem

The problem of airline passenger flow prediction can be described as follows: the historical flow data of each station *X*_*t*−*s*_, *X*_*t*−*s*+1_, *X*_*t*−2_, ⋯ , *X*_*t*−1_ (s is the time step) can be used to predict the flow *X*_*t*_ of the next period. The formula is described as

(1) $$\begin{array}{r} X_{t}=F\left(\left[X_{t-s},X_{t-s+1},\cdots \;,X_{t-2},X_{t-1}\right]\right), \end{array}$$

where, *X*_*t*_ is the site characteristics at each time step, and *F* is a nonlinear function.

In the actual traffic system, the network is regarded as a directed graph *G* = (*Q, V, A*). Each sensor in the network is regarded as a node *v*_*i*_ and its value *Q* ∈ *R* is a scalar. *V* ∈ *R*^*N*^ and *N* is the number of sensors. The flow relationship between nodes consists of adjacency matrix *A* that is, the element *A*_*ij*_ in *A* represents the connection relationship between node *V*_*i*_ and *V*_*j*_.

### 2.2. The Description of the GCN

When dealing with the structure of the graph, it is necessary to obtain its Symmetric normalized Laplacian matrix *L*, which is generally defined in the following ways:

(2) $$\begin{array}{r} L=D^{-\frac{1}{2}}\left(D-A\right)D^{-\frac{1}{2}}=I_{N}-D^{-\frac{1}{2}}AD^{-\frac{1}{2}}, \end{array}$$

where, *I*_*N*_ is the identity matrix of *N* × *N*; Degree matrix *D* is defined as $D_{ii}=\underset{i}{\sum }A_{ij}^{i}$. Decompose the eigenvalue of *L* to get *L* = L = UΛU^T^. Λ is made up of L eigenvalues of diagonal matrix. U = {u_1_, u_2_, …, u_N_} is composed of the eigenvector L, and it is an orthonormal basis for R^N^.

The spectral convolution theory in the graph structure has been supplemented and perfected in the paper. The convolution operation of convolution kernel *G* and input signal *X* in the time domain can be converted into the inner product form in the frequency domain.

(3) $$\begin{array}{r} g^{*}x=U\left(U^{\text{T}}g\right)⊙\left(U^{\text{T}}x\right))=U_{g_{\theta }}\left(A\right)U^{\text{T}}x, \end{array}$$

where $g_{s}\left(\Lambda \right)=U^{T}g=\text{diag}\left(\theta \right),\theta \in R^{N}$, ⊙ represents the hadamar product, *U*^*T*^*g* means mapping *g* to the frequency domain space based on *U*. Due to *g*_θ_ high computational complexity, so hierarchical linear model constraints and the chebyshev polynomial are used to approximate calculation. In this paper, the simplified first order polynomial form of g*x is adopted.

(4) $$\begin{array}{r} g^{*}x=U_{g_{\theta }}U_{x}^{\text{T}}\approx \theta \left(I_{N}+D^{-1/2}AD^{-1/2}\right)x, \end{array}$$

where ${\tilde{D}}^{-1/2}\tilde{A}{\tilde{D}}^{-1/2}=I_{N}+D^{-1/2}AD^{-1/2}\;Ã=I_{N}+A$, $D=\underset{i}{\sum }{\tilde{A}}_{ij}$. Therefore, the output of layer *L* is

(5) $$\begin{array}{r} H^{\left(l\right)}=\sigma ({\tilde{D}}^{-\frac{1}{2}}\tilde{A}{\tilde{D}}^{-\frac{1}{2}}H^{\left(l-1\right)}W^{\left(l\right)}, \end{array}$$

where δ is the activation function, ${\tilde{W}}^{\left(l\right)}=\theta^{\left(l-1\right)}W^{\left(l\right)},\;\theta^{\left(l-1\right)}\in R^{c^{\left(1-l\right)}\times F^{\left(l-1\right)}},W^{\left(l\right)}\in R^{F^{\left(l-1\right)}\times c^{\left(l\right)}}$, *C*^(*L* − 1)^ is the output dimension of the (*L* − 1) layer, and *F*^(*L*−1)^ is the characteristic vector size of each dimension. Therefore

(6) $$\begin{array}{r} H^{\left(l\right)}=\sigma \left({\tilde{D}}^{-\frac{1}{2}}\tilde{A}{\tilde{D}}^{-\frac{1}{2}}H^{\left(l-1\right)}{\tilde{W}}^{\left(l\right)}\right). \end{array}$$

At present, there is no effective measurement method for the calculation of adjacency matrix *A*. Most scholars use heuristic methods, that is, based on the Euclidean distance or Markov distance between sensors to determine the element value corresponding to the adjacency matrix. However, these methods all require manual calculation of the distance relationship between the sensors in advance. In this paper, the data-driven method is adopted to calculate the adjacency matrix, and *A* = *D*. The formula can be written as

(7) $$\begin{array}{r} H^{\left(l\right)}=\sigma \left(\tilde{A}H^{\left(l-1\right)}{\tilde{W}}^{\left(l\right)}\right). \end{array}$$

The element value of matrix *A* is learned from the sample data, that is, the matrix is composed of trainable parameters. The data-driven approach is more realistic than the heuristic approach. Therefore, the L layer of the convolutional neural network is constructed in accordance with Formula 7. It should be noted that the initial matrix *A* is the same for each layer of the convolutional network, and the parameters are updated only when the error is propagated backwards.

GCN introduces the spatial features of the graph by convolving the Laplace matrix with the input. In this paper, the model takes flight segments as nodes and the association between flight segments as edges to build a graph. According to the graph, the adjacency matrix is obtained and the demand of future flight segments is predicted by combining the price and demand of historical flight segments.

### 2.3. The Problem of Time Series

It is found that Recurrent Neural networks (RNN) are widely used in sequential data such as natural language and image processing, which have a significant effect. Since then, various types of Circulating Neural Networks have been used. Aiming at the problem of air passenger flow prediction, this paper introduces the LSTM, which can extract the characteristic information of the input sequence and find its internal relation, so as to improve the prediction accuracy of the model. In order to make use of the spatial-temporal characteristic of the airborne data information at the same time, the GCN model is combined with the LSTM model, and it is added to the output of the upper level.

**Figure 2:**
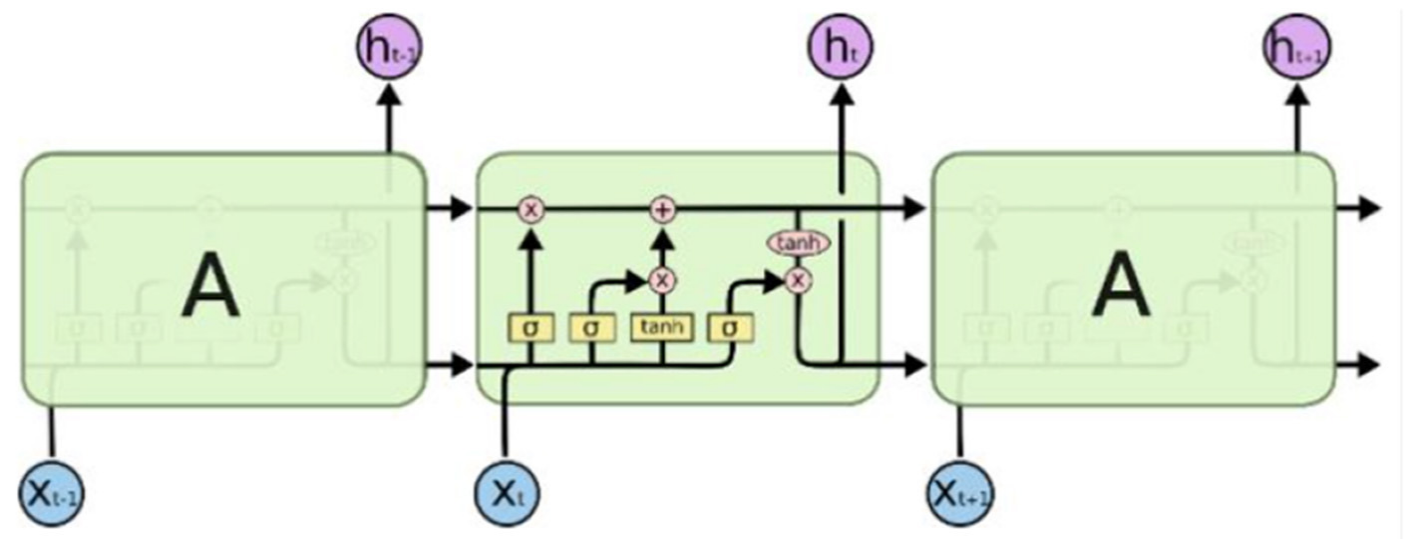
The structure of LSTM.

The LSTM network structure used in this paper is shown as Figure 2.

The network model mainly accepts three inputs: *X*, *H* and *C* represent the current state, hidden layer state and cell state, respectively.

LSTM mainly realizes the management of long and short term memory through three gating units. Firstly, the LSTM needs to determine what information needs to be thrown out. This step is decided by the layer called the “Forget Gate.” Input *x* and *h*, output a number between 0 and 1. The value of 1 means “keep the value completely,” while 0 means “throw the value away completely.” The formula of forgetting gate is as follows:

(8) $$\begin{array}{r} f_{t}=\sigma \left(W_{f}\cdot \left[h_{t-1},x_{t}\right]+b_{f}\right), \end{array}$$

where *W*_*f*_ and *b*_*f*_ are the parameters to be learned, and σ is the sigmod activation function.

Secondly, the LSTM needs to determine what information need to store in the cell state. There are two stages to this question. First, the layer of “Input Gate” determine which data needs to be updated. Then, the vector *C*_1_ was created by a tanh layer.

(9) $$\begin{array}{r} i_{t}=\sigma \left(W_{i}\cdot \left[h_{t-1},x_{t}\right]+b_{i}\right),\\ {\tilde{C}}_{t}=\tanh\left(W_{C}\cdot \left[h_{t-1},x_{t}\right]+b_{C}\right). \end{array}$$

After deciding what needs to be forgotten and what needs to be added, *C*_*t*−1_ can be updated to *C*_*t*_.

(10) $$\begin{array}{r} C_{t}=f_{t}*C_{t-1}+i_{t}*{\tilde{C}}_{t}. \end{array}$$

Finally, we need to decide what to export. This output is based on our cell state. The final output is part of the cell state. First, we run an output gate to determine which part of the cell state we are going to output. Then we put the cell state into the tanh (pressing the value between –1 and 1). Finally we multiply it by the output of the output gate.

(11) $$\begin{array}{l} \begin{array}{c} o_{t}=\sigma \left(W_{o}\left[h_{t-1},x_{t}\right]+b_{o}\right),\\ h_{t}=o_{t}*\tanh\left(C_{t}\right). \end{array} \end{array}$$

### 2.4. The Description of Algorithm

**Figure 3:**
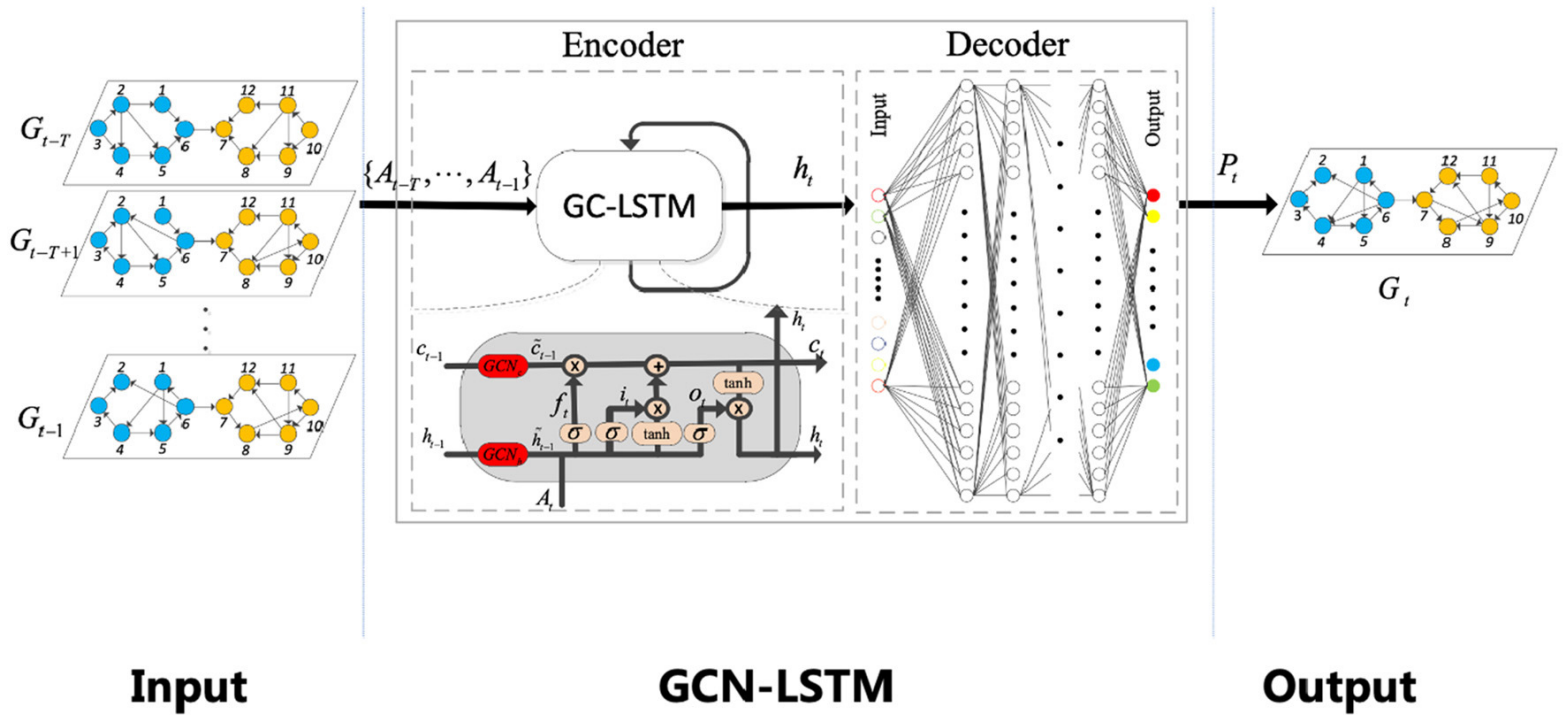
Overall structure of GCN-LSTM model (original features of air passenger flow data are extracted by using first-order approximate GCN, and output features are analyzed by LSTM for long-term and short-term sequence characteristics, and then predicted values are obtained).

The network structure based on GCN-LSTM model proposed in this paper is shown in the Figure 3. The model mainly adopts encoder-decoder structure. In the encoder, multiple parallel GCN modules are used to extract the key features of the graph network with different time series. Then, the extracted time series features are transmitted to LSTM, and feature analysis and further feature extraction are carried out on the sequence data through LSTM to solve the long-term and short-term dependencies between the data. Finally, the encoder generates an encoded pair vector and sends it to the decoder. In the decoder, the multi-layer feedforward neural network is used to further process the features of the coding vector. Finally, the processed data is transmitted to a GCN network to produce predicted values.

In order to improve the prediction performance of the GCN-LSTM algorithm, in this paper, we use the final output through the network and the value of the real label to calculate *L*_1_ loss, also known as the mean absolute error (MAE), and take the mean square error (MSE) as the evaluation index of the model. The specific calculation formula is as:

(12) $$\begin{array}{r} \mathrm{loss}=MAE=\frac{1}{m}\overset{m}{\underset{i=1}{\sum }}|\left(y_{i}-{ŷ}_{i}\right)|.\\ MSE=\frac{1}{m}\overset{m}{\underset{i=1}{\sum }}{\left(\left(y_{i}-{ŷ}_{i}\right)\right)}^{2}. \end{array}$$

In this paper, the Adam-based batch gradient descent optimization algorithm is used to learn and update parameters through the loss function to minimize the loss function until the loss function converges. The trained model is used to predict the test set and calculate the MSE. The smaller the MSE value is, the closer the predicted value of the model is to the real value and the better the generalization ability of the model is.

## 3. Experiments

This paper selects 6, 9111, 332 ticket sales data examples of 28,809 flights of 17 domestic airlines between September 1, 2020 and October 31, 2020 to verify the feasibility and effectiveness of the GCN-LSTM combined prediction model. The selected data set is relatively complete without missing values. After obtaining a complete data set, an adjacency matrix is constructed according to the data set. Each flight segment is a node. Between nodes, if the origin is the same, it is considered to have a diverting effect on traffic, and the weight is set as the number of (0,1). If the destination is the same and the traffic is considered to be promoted, the weight is set as the number greater than 1 and the rest as 0.

In order to do a comparative experiment, our experimental environment and experimental parameters are set as: epoch = 100, batch size = 1,000, learning rate = 0.001, GPU = NVIDIA 2080Ti. The ratio of training set to test set is 8:2.

Five sections of AAT_URC, CAN_PEK, CAN_CSX, CAN_CTU, and CAN_CKG are taken as examples to construct an adjacency matrix, in which AAT, URC, CAN, PEK, CSX, CTU, and CKG are city names. The adjacency matrix of these five cities is shown in Table 1. If the origin of two flight sections is the same, it is considered to have a diversion effect on traffic, and the weight is set as 0.5. None of the five sections had the same destination, so the rest of the weights were set to 0.

**Table 1:** Adjacent matrix.

	**AAT_URC**	**CAN_PEK**	**CAN_CSX**	**CAN_CTU**	**CAN_CKG**
AAT_URC	0	0.0	0.0	0.0	0.0
CAN_PEK	0	0.0	0.5	0.5	0.5
CAN_CSX	0	0.5	0.0	0.5	0.5
CAN_CTU	0	0.5	0.5	0.0	0.5
CAN_CKG	0	0.5	0.5	0.5	0.0

Then we need to construct a time series of prices. The above five flight sections are also taken as examples. According to the take-off time, the price series 14 days before the take-off time is taken as the *X* feature. The flow of 1 day before the take-off was taken as *Y*, and such input and output were a sample. The take-off time was calculated forward to increase the number of samples. In this way, there are 14 days' data, one piece of demand data, arranged in a time series according to time.

In this paper, the data set of 2 months is divided into training set and test set according to the ratio of 8:2, normalized and trained by GCN-LSTM. The timing step size of the model is 15, and the window is constantly moved to predict. Under the premise of the same input of historical price time series, it is compared with the experimental results of autoregressive model (AR), Moving Average(MA), Exponential Smoothing(ES), Long short-term memory(LSTM) and Support Vector Regression(SVR).

Table 2 lists five precise results of route prediction, The prediction mean square error (MSE) of the proposed GCN-LSTM model is smaller than that of LSTM, SVR, AR, MA and ES. The results show that the GCN-LSTM model can improve the accuracy of prediction.

**Table 2:** Prediction results of different methods.

	**GCN-LSTM(%)**	**LSTM(%)**	**SVR(%)**	**AR(%)**	**MA(%)**	**ES(%)**
Average of five routes	6.86	10.84	12.72	14.46	13.73	16.93
AAT_URC	5.44	8.71	10.73	11.37	10.74	14.39
CAN_PEK	6.91	9.74	12.02	12.75	11.71	16.47
CAN_CSX	8.87	12.03	13.07	17.13	14.57	18.29
CAN_CTU	6.24	11.73	14.35	15.01	13.79	17.48
CAN_CKG	6.84	11.99	13.43	16.04	17.84	18.02

**Figure 4:**
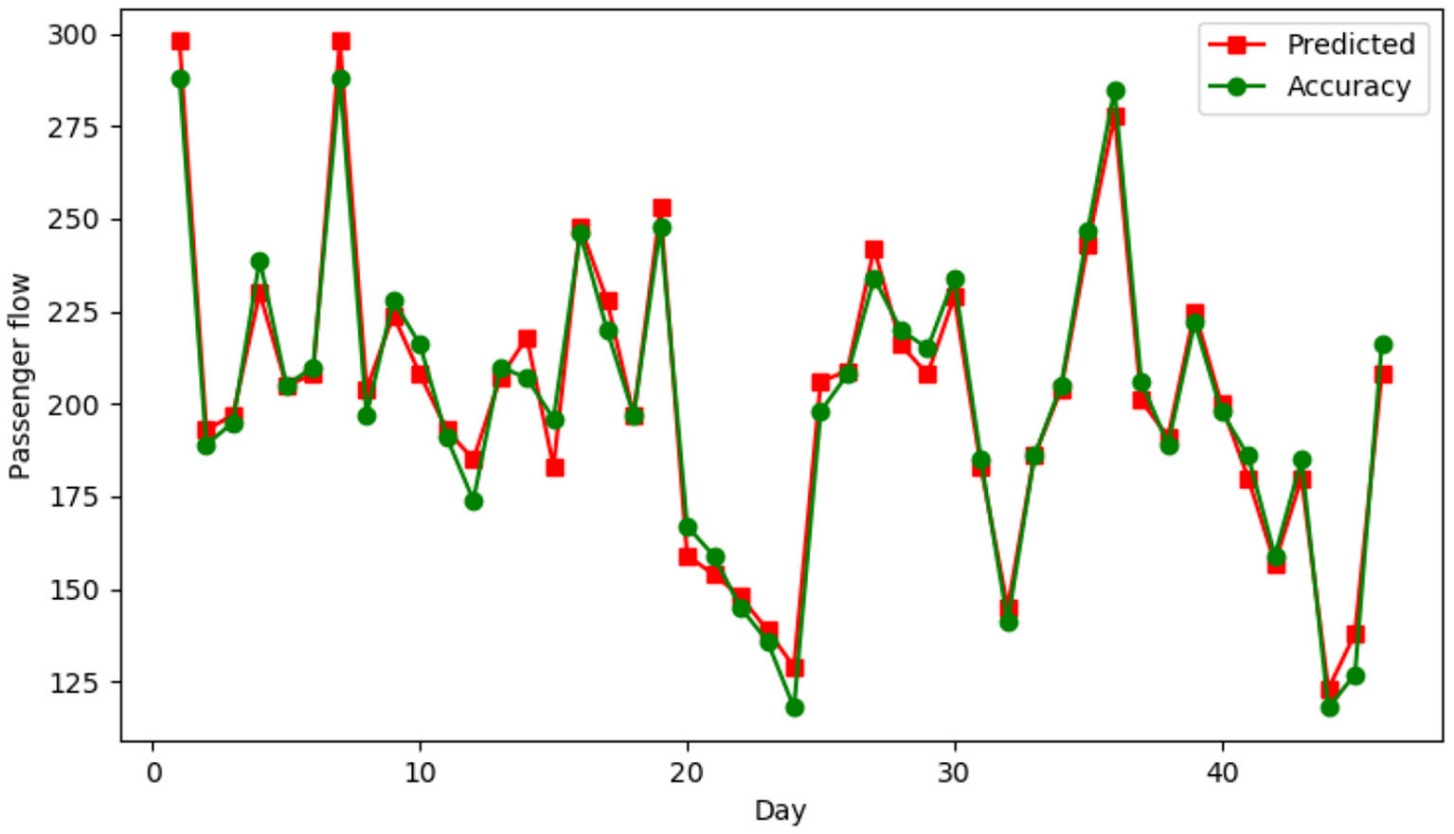
OGCN-LSTM model prediction results of AAT URC route.

AAT_URC a single route, for example, using the proposed GCN-LSTM traffic prediction model, the horizontal axis shows departure date from September 15, 2020 to October 31, 2020, 14 days before the use of the time sequence to forecast the traffic on the same day price, compare the renderings as shown in Figure 4, dot shows the actual passenger flow, square said according to 14 days before the price time series prediction of passenger flow.

**Figure 5:**
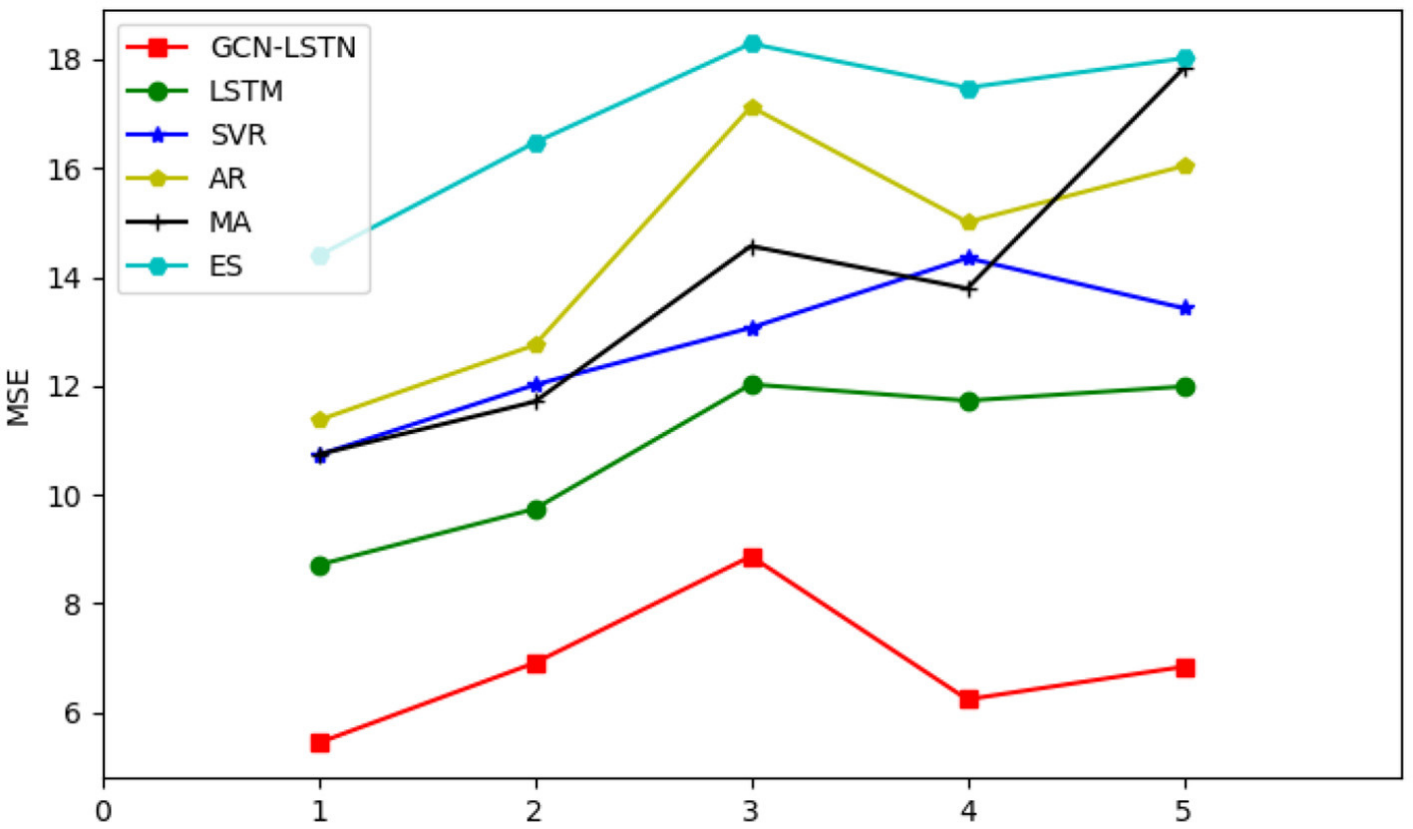
Comparison of MSE results of five airlines.

Figure 5 shows the average MSE values predicted by AR model, moving average, exponential smoothing, LSTM and SVR for 5 airlines. The horizontal axis represents five different airlines, the vertical axis represents square error (MSE), and the red line is the prediction results of the proposed GCN-LSTM model.

It can be seen from the figure that the GCN-LSTM model is used for prediction, and the error fluctuation is small, and the MSE is basically in the range of 5%-9%. The prediction results of the proposed GCN-LSTM model are obviously better than those of other models.

## 4. Conclusion

In view of the problems existing in air flow prediction, an air flow prediction model based on graph convolutional neural network and long short-term memory network is proposed based on in-depth analysis of the influencing factors of air flow prediction. Firstly, based on the characteristics of air traffic data, a feature extraction network based on graph convolutional neural network is designed. Then combined with the long—short memory network to solve the problem of long—short—term data dependence; Finally, the prediction results are outputted based on the feedforward neural network. In the experimental part, we verify the performance of the GCN-LSTM model on aviation data sets. The experimental results show that the training effect of this model is obviously better than other models, and it has lower MSE loss value. This also shows that the prediction result has a higher prediction accuracy.

## Data Availability Statement

The datasets presented in this study can be found in online repositories. The names of the repository/repositories and accession number(s) can be found below: https://github.com/qetmes/GCN-LSTM.

## Author Contributions

The author confirms being the sole contributor of this work and has approved it for publication.

## Conflict of Interest

The author declares that the research was conducted in the absence of any commercial or financial relationships that could be construed as a potential conflict of interest.

## Publisher's Note

All claims expressed in this article are solely those of the authors and do not necessarily represent those of their affiliated organizations, or those of the publisher, the editors and the reviewers. Any product that may be evaluated in this article, or claim that may be made by its manufacturer, is not guaranteed or endorsed by the publisher.
